# The relationship between climate change anxiety and pro-environmental behavior in adolescents: the mediating role of future self-continuity and the moderating role of green self-efficacy

**DOI:** 10.1186/s40359-024-01746-1

**Published:** 2024-04-27

**Authors:** Ziqi Qin, Qi Wu, Cuihua Bi, Yanwei Deng, Qiuyun Hu

**Affiliations:** School of psychology, Sichuan Normal University, Sichuan, 610066 China

**Keywords:** Climate change anxiety, Pro-environmental behavior, Future self-continuity, Green self-efficacy

## Abstract

**Background:**

Climate change is seriously affecting human survival and development, and the anxiety caused by it is becoming increasingly prominent. How to alleviate people’s climate change anxiety, improve the ecological environment, and promote the formation of green lifestyles among people, especially young people, is an important topic that deserves to be explored. This study examined the relationship between climate change anxiety and pro-environmental behaviors and the underlying psychological mechanism in the adolescents.

**Methods:**

This study explored the crucial role of future self-continuity (FSC) between climate change anxiety (CCA) and pro-environmental behaviors (PEB) in adolescents and examined the moderating role of green self-efficacy (GSE). In this study, a total of 1,851 middle and high school students from five schools were selected for questionnaire survey.

**Results:**

The results showed that (1) in both middle and high school grades, there was a significant negative correlation between climate change anxiety and pro-environmental behaviors; future self-continuity was significantly positively correlated with pro-environmental behaviors; green self-efficacy was negatively correlated with climate change anxiety and positively correlated with pro-environmental behaviors; (2) climate change anxiety negatively predicted pro-environmental behaviors, and compared with middle school grades, high school grade adolescents’ climate change anxiety was significantly predicted pro-environmental behaviors. Future self-continuity mediated the relationship between climate change anxiety and pro-environmental behaviors in both grades. (3) green self-efficacy moderated the second half of the pathway of the mediation model only in middle grades. Specifically in middle school, future self-continuity did not significantly predict pro-environmental behaviors at low green self-efficacy level, but positively predicted pro-environmental behaviors at high green self-efficacy level. In high school, future self-continuity did not significantly predict pro-environmental behaviors in either high or low green self-efficacy level.

**Conclusion:**

This study suggests that there is a moderated mediation model between adolescents’ climate change anxiety and pro-environmental behaviors, with different mediating and moderating effects among adolescents in various grades. This is of great significance in alleviating climate anxiety among adolescents and cultivating their pro-environmental behaviors.

## Introduction

### Relationship between climate change anxiety and pro-environmental behaviors

A report published in the top international medical journal The Lancet stated that global temperatures reached their highest point in 100,000 years in 2023, with high temperature recorded broken on every continent, causing fatal damage to human health worldwide. According to the *Global Risks Report 2023* published by the World Economic Forum, the failure to mitigate climate change and to adapt to climate change have already been ranked as the top two global risks in terms of severity over the next decade. The world’s environmental problems are jeopardizing the survival and development of human. Negative emotions have been triggered by the concerns about the consequences, such as anxiety and distress, which can affect physical and mental health. In a global survey of 10,000 young people aged 16–25, nearly 60% of respondents said they were “extremely worried” or “apprehensive” about climate change, and 75% said that “the future is scary” [1]. The Stress-Vulnerability Model of Mental health demonstrated that this may lead to measurable structural or functional changes in the brain and psychopathology later in life, if children and adolescents still frequently exposure to stressors during the formative years [2–4].

Climate change affects mental health in three main ways: direct, indirect, and alternative. Most studies mainly focus on the direct influences, such as post-traumatic stress disorder, depression, anxiety, suicide, etc., caused by extreme weather events liking floods, earthquakes, or hurricanes [5–7]. Indirect way refers to the influences by the economy, migration, destruction of social infrastructure, and shortage of food and water resources [8]. Alternative way means any effect on individuals due to climate change information by use of modern communication technology [9]. In the learning process, the source of information may be equally important or even more important than the information itself [10]. Research has shown that more environmental knowledge is significantly correlated with higher levels of environmental behavior [11]. According to *the Youth Blue Book: Report on Internet Use of Chinese Minors in 2023*, the Internet penetration rate of minors is almost saturated, significantly higher than the national Internet penetration rate (75.6%). The proportion of internet users under the age of 10 and those aged 10–19 is 4.4% and 14.3%, respectively. Moreover, the number of young Internet users in China has reached nearly 200 million. Previous study has found that the Internet is a major source of information for adolescents, and that as individuals get older, they acquire more information and knowledge about climate change [12].

Climate Change Anxiety, also known as ecological anxiety, refers to people’s fear of the changing and uncertain natural environment [13]. Climate change anxiety can affect people’s cognitive, emotional, decision-making, and behavioral responses, such as persistent concerns, psychological distress, sleep difficulties, and even affect cognitive deficits, learning, adaptation, and interpersonal relationships [14–18]. Moreover, warmer temperatures can impair mental health and increase the risk of suicidal behavior [7, 19]. Pro-environmental behavior, also known as green behavior, is essentially concerned with behaviors that are beneficial to the environment or at least minimize negative impacts on the environment at the individual or family level [20]. This kind of behavior can reduce ecological damage, protect natural resources, and improve environmental quality, including the private realm (e.g. green consumption, low-carbon travel) and the public realm (e.g. supporting environmental policies and donating to environmental organizations) [21].

There is a complex relationship between climate change anxiety and pro-environmental behaviors. Some studies have shown that climate change anxiety plays a positive and constructive role in improving pro-environmental behavior [22–24]. On the contrary, other studies showed that the negative psychology generated by climate change can hinder individuals from dealing with problems [25, 26]. Specifically, climate change anxiety may weaken an individual’s reaction capability, leading to feelings of helplessness and despair. This condition is known as ecological paralysis [27], characterized by emotional manifestations such as depression, excessive anxiety, despair, and apathy. That’s to say, when faced with malignant climate change, individuals tend to adopt a more indifferent attitude rather than pro-environmental behavior [28].

An Australian study investigated the trajectory of climate worries among adolescents over an eight-year period (2009–2010 and 2017–2018), and reported that there was an increase in adolescent concerns about climate issues over time [29]. On the one hand, research has shown a positive correlation between individual responsibility and environmental willingness, and enhancing individual responsibility can increase students’ pro-environmental behaviors [30]. Meanwhile, the study also found that environmental self-efficacy and intergenerational obligations can positively predict the pro-environmental behavior of young people [31]. However, on the other hand, for most children, they may have a harder time than adults dealing with the negative emotions caused by such threats [32], struggling to cope in a constructive way, and more prominently displaying apathy, and denial. This can be explained by the fact that young and frail teenagers may subjectively think that they have no responsibility to protect the environment, and are more willing to believe that some more capable people or institutions, such as the government, should take action to deal with the threat of climate change [1]. Based on the above analysis, this study proposes Hypothesis 1: Climate change anxiety is negatively correlated with pro-environmental behavior.

### The mediating role of future self-continuity

The most basic time dimension of climate change may be its extension to the future [33]. When looking to the future, people tend to positively view the future, believing that the future is better than the present. According to the theory of self-perception and self-determination, an individual’s self-awareness is not only limited to the present self, but also associated with their past and future. Future self-continuity refers to the degree of close connection between the present and future selves [34]. Future self-continuity is a temporal dimension of self-representation in which people can imagine the future, in this way people can perceive the event as being closer in time [35, 36]. The closer the psychological connection between the present self and the past or and future, the stronger the emotional response experienced, and the easier it is to motivate individuals to make more visionary behaviors [37]. How people perceive their future “selves” will affect intertemporal decision making and various aspects of behavior, such as expenditure, academic performance, and prosocial behavior. Future Self-Continuity Model states that increasing the connection between an individual’s future self and present self will lower the time discount rate and make individuals more willing to wait for more rewards in the future [38]. For example, a high degree of future self-continuity more likely to control current expenditures and save money [39]. Conversely, individuals with low future self-continuity perceive their future selves as different from their present selves and are less likely to think forwardly.

Using future situational thinking to experience climate change in advance will enhance people’s risk perception of climate change, thereby promoting the trend of taking pro-environmental actions [40]. The study of Zaval, Markowitz, and Weber reported [41], if people could focus on their legacy, they would increase their subsequent donations to environmental charities, demonstrate greater willingness to be environmentally friendly, and have a greater confidence in climate change. The research of Syropoulos and Markowitz suggests that an individual’s sense of responsibility towards future generations is a powerful predictor of their pro-environmental behavior, especially in the context of addressing global climate change [42]. Stronger generative concern in emerging adulthood positively predicted environmentalism [43]. Long termists have the ability to imagine a brighter and more sustainable future, which allows them to avoid being influenced by other factors when paying attention to the environment, and to play an important role in shaping individual sustainable actions [44].

Anxious individuals have increased negative expectations about the future and may overestimate the likelihood of harmful consequences that may occur in the future [45, 46]. And increased anxiety may lead to less concrete thoughts about the future [47]. It has been shown that adolescents with higher levels of anxiety have more negative feelings about the past, present, and future [48]. Based on these analyses, future self-continuity can explain the mechanisms underlying climate change anxiety and pro-environmental behaviors, higher levels of future self-continuity may help adolescents focus on the future, linking the present self more closely to the future self [49]. Therefore, this study proposes Hypothesis 2: Future self-continuity plays a mediating role between climate change anxiety and pro-environmental behavior.

### The moderating role of green self-efficacy

Whether individuals can successfully and continuously implement environmental behaviors is related to green self-efficacy. The definition of green self-efficacy is the level of confidence an individual possesses in planning, executing, and completing environmental goals and tasks [50]. According to the Environmental Perceived Stress Model (EPSM), in the face of climate change, such as global warming, people’s assessment of their ability will determine their problem-solving, self-protection and emotional response behaviors [51]. Green self-efficacy promotes green consumption behavior [52] and influences green performance, green creativity, pro-environmental behavior, and green purchase intention [53–56].

Self-efficacy is a subjective judgement based on one’s own knowledge and experience, which affects the way a person thinks and behaviors [57], and thus is often used as a moderating variable in the psychological responses to perceived feelings [58]. It has been demonstrated that green self-efficacy plays a positive moderating role between perceptions of green organisational support and employees’ green innovation behaviors [59]; Faraz et al. found that green servant leaders influence environmental behaviors through green self-efficacy [60]. Jerusalem and Mittag pointed out that individuals with higher self-efficacy believe that they have the ability to control the current situation [61], and behave more positively when confronted with problems, usually attributing positive results to their own efforts and negative results to external uncertainties. While individuals with lower self-efficacy often have self-doubt and feel that they are unable to cope with the current situation, which leads to psychological problems such as anxiety and worry. Self-efficacy is a protective factor for psychological well-being [62], and individuals with high self-efficacy are able to better handle their negative emotions, and positively respond to problem solving and challenges. Based on the above analysis, we proposed Hypothesis 3: Green self-efficacy plays a moderating role between climate change anxiety and pro-environmental behavior. Specifically, when the adolescents’ green self-efficacy is higher, pro-environmental behavior are more speculated by future self-continuity.

### Effect of age

Adolescence is a unique period of cognitive development in the brain, with functions such as impulse control, memory, emotional regulation, and decision-making [63–65]. The impact of age on pro-environmental behavior is inconsistent. Middle-aged and older adults are more concerned about nature and ecology-related issues than younger adults, and are more likely to engage in pro-environmental behaviors [66]. Similar research findings also indicate that high school adolescents exhibit fewer pro-environmental behaviors than lower grade adolescents [67]. Yet some researchers suggest that young people are more concerned about environmental issues and have a higher frequency of pro-environmental behaviors such as using public transportation and recycling behaviors [68–71]. Therefore, this study will explore whether there are differences at higher and lower grade levels in the relationship between climate change anxiety and pro-environmental behaviors.

**Fig. 1:**
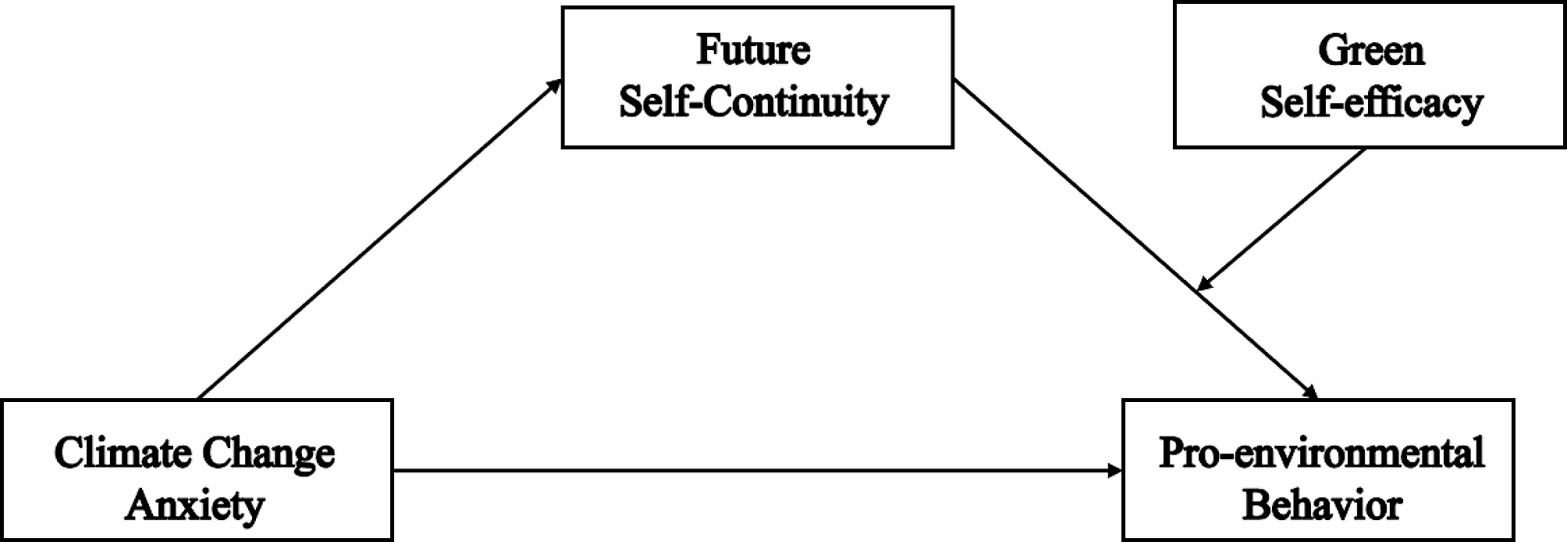
The conceptual moderated mediation model

Previous studies have examined the effects of institutional, economic, and environmental knowledge on pro-environmental behaviors, and explored the role of collective and individual self-efficacy in promoting pro-environmental behaviors [72, 73], focusing on the environmental domain, green self-efficacy plays a more unique role. However, existing research have not examined the role that future self-continuity plays in the relationship between climate change anxiety and pro-environmental behavior [74]. Moreover, previous studies have not focused on the adolescent population and have mostly studied the larger population of citizens. Therefore, this study aimed to investigate the relationship between climate change anxiety and pro-environmental behaviors in a group of adolescents, and explored the mediating and moderating roles of future self-continuity and green self-efficacy in adolescents of different ages. As shown in Fig. 1.

## Methods

### Participants and procedures

Two high schools and three middle schools in Chengdu, Sichuan Province, China, were randomly selected. A total of 2,000 questionnaires were distributed and 1,900 were collected, with a recovery rate of 95%. Due to incomplete and single answers, 49 invalid questionnaires were excluded, a total of 1,851 valid questionnaires were obtained. The socio-demographic information of the participants is shown in Table 1. There was no pre-registration for this study.

Considering that middle school students and high school students mainly study in school and do not carry mobile phones, all questionnaires are distributed in paper versions in class. Prior to the questionnaire survey, informed consent was obtained from students, parents, and school administrators. The investigative process was supported by psychological teachers and class teachers. The study has been approved by the Ethics Review Committee of Sichuan Normal University.

**Table 1:** Participant characteristics

Variables	Category	*N*	Percentage(%)
Gender	Male	822	44.41%
	Female	1029	55.59%
School	High school 1	430	23.23%
	High school 2	431	23.28%
	Middle school 1	270	14.59%
	Middle school 2	219	11.83%
	Middle school 3	501	27.07%
Student’s Place of Residence	Urban	1671	90.28%
	Rural	180	9.72%
Residency	Reside at school	757	40.90%
	Not-resident	1094	59.10%
Unique births	A single birth	758	40.95
	Not a single birth	1093	50.05%
Parental Marital Status	Divorce	244	13.18%
	Non-Divorced	1607	86.82%

### Measures

#### Climate change anxiety

The Climate Change Anxiety Scale (CCAS), developed by Clayton and Karazsia in 2020 [14], is used to measure the level of climate change anxiety among adolescents. Previous studies have shown that this measurement tool widely used in different countries for climate change anxiety has similar structures and psychological meanings, making this climate change anxiety scale suitable for the Chinese population [75]. The scale is divided into two dimensions, cognitive-emotional impairment, and functional impairment, and consists of 13 question items with no reverse scoring items; higher scores indicate higher levels of climate change anxiety. The Cronbach’s alpha coefficient in this study was 0.91.

#### Pro-environmental behavior

This study used the Pro-Environmental Behavior Questionnaire (PEBQ) revised by Kaiser et al. in 2007, and to select appropriate questions for measuring pro-environmental behavior [76]. Research suggests that due to the different international backgrounds of social policies and environmental issues, the pro-environmental behavior questionnaire exhibits different functions and issues when used in different countries. In addition, some items in the scale may not be in line with the actual behavior of adolescents, resulting in relatively low reliability (0.74) [77]. The questionnaire consists of 15 items (e.g., “Separating garbage into different categories”). It is scored on a 5-point scale, five of which are reverse scored, with higher total scores representing more pro-environmental behaviors. The Cronbach’s alpha coefficient in this study was 0.76.

#### Future self-continuity

Future Self-Continuity Questionnaire (FSCQ) was developed by Sokol and Serper [78]. The Chinese version was tested by Zhang Feng and others for good reliability and validity [79]. The questionnaire consists of three dimensions, namely similarity, vividness and positivity. There are a total of 10 items, with a 6-point scoring system. The higher the total scores, the higher the level of future self-continuity. The Cronbach’s alpha coefficient in this study was 0.86.

#### Green self-efficacy

Green Self-efficacy Questionnaire was revised by Du et al. in 2022 to measure the level of individual green self-efficacy [80]. The questionnaire consists of four dimensions, including environmental responsibility (e.g., “It is my responsibility to do my best to protect the environment and conserve resources.“), green self-efficacy (e.g., “I feel that I can successfully practice environmental protection concepts.“), perceived value of green (“Using green products helps improve the ecological environment.“), green purchase intention (“I am willing to look for and buy green products.“). There are a total of 17 items, with a 7-point scoring system, all of which are positive scoring. The Cronbach’s alpha coefficient in this study was 0.97.

### Data analysis

This study used SPSS 26.0 for common method testing, multiple linear regression, descriptive statistics, and correlation analysis, as well as using the PROCESS 4.0 plug-in to test the moderated mediation model.

#### Control variables and Covariance

This study controlled of gender (0 = male, 1 = female) and age. Tolerance (Tol) of data results for multiple linear regression analysis ranged from 0.847 to 0.970, all greater than 0.1, and Variance Inflation Factor (VIF) ranged from 1.031 to 1.181, all less than 10. The data results indicate that there is no multicollinearity in the variables of this study.

## Results

### Test for common method bias

The Harman one-way test was used to test common method biases. The results showed that there were 9 factors with their characteristic root greater than 1. The first factor explaining 19.53% of the cumulative variance, which is less than the threshold of 40%, indicating that there were no serious common method biases.

### Homogeneity test

In order to ensure whether pro-environmental behaviors were influenced by gender and number of children, the independent samples t-test was used. The result showed that there was no difference in the scores of pro-environmental behaviors between male and female (*t*_1849_ = 0.73, *p* = 0.46 < 0.001); regardless of whether they are only child or not, the scores of pro-environmental behaviors also had no significant difference (*t*_1849_ = 0.932, *p* = 0.75 > 0.001).

ANOVA was used to test whether there were differences in pro-environmental behaviors between schools, and it was showed that the main effect of school was significant (*F*(4, 1850) = 56.50, *p* < 0.001). Post hoc tests showed that there was no significant difference between the two high school schools, and among the three middle school schools. Therefore, we coded them as high school and junior high school, and conducted independent samples t-tests. The results indicated that there were significantly more pro-environmental behaviors in the middle school (*M* = 3.67, *SD* = 0.69) than in high school schools (*M* = 3.22, *SD* = 0.59), *t*_1849_ = 15.08, *p* < 0.001. As a result, the subsequent mediation analysis was tested separately at the middle and high school levels.

### Multivariate analysis results

Correlation analysis was shown in Table 2. Correlation analyses using the mean scores of each variable revealed that CCA was negatively associated with FSC (Middle: *r*=-0.16, *p* < 0.01; High: *r*=-0.12, *p* < 0.01) and PEB (Middle: *r*=-0.12, *p* < 0.01; High: *r*=- 0.09, *p* < 0.01). Also, CCA was negatively associated with GSE (Middle: *r*=-0.14, *p* < 0.01; High: *r*=-0.10, *p* < 0.01). There was positive correlation between FSC and GSE (Middle: *r* = 0.42, *p* < 0.01; High: *r* = 0.28, *p* < 0.01) as well as FSC and PEB (Middle: *r* = 0.30, *p* < 0.01; High: *r* = 0.36, *p* < 0.01). GSE was positively correlated with PEB (Middle: *r* = 0.56, *p* < 0.01; High: *r* = 0.46, *p* < 0.01).

**Table 2:** Descriptive results and correlation analysis of variables

		M	SD	1	2	3	4
1. Future Self-Continuity(FSC)	Middle school	4.12	0.90	1			
	High school	3.87	0.81	1			
2. Green Self-efficacy(GSE)	Middle school	5.89	1.12	0.42^**^	1		
	High school	5.23	1.09	0.28^**^	1		
3. Climate Change Anxiety(CCA)	Middle school	1.57	0.61	-0.16^**^	-0.14^**^	1	
	High school	1.67	0.65	-0.12^**^	-0.10^**^	1	
4.Pro-environmental Behavior(PEB)	Middle school	3.71	0.63	0.30^**^	0.56^**^	-0.12^**^	1
	High school	3.37	0.56	0.36^**^	0.46^**^	-0.09^**^	1

Note.: *p < 0.05, ^**^p < 0.01, ^***^p < 0.001. The variables in the model are standardised; all values are retained to two decimal places. FSC means Future Self-Continuity; GSE means Green Self-efficacy; CCA means Climate Change Anxiety; PEB means Pro-environmental Behavior

### Mediating effects of future self-continuity

**Fig. 2:**
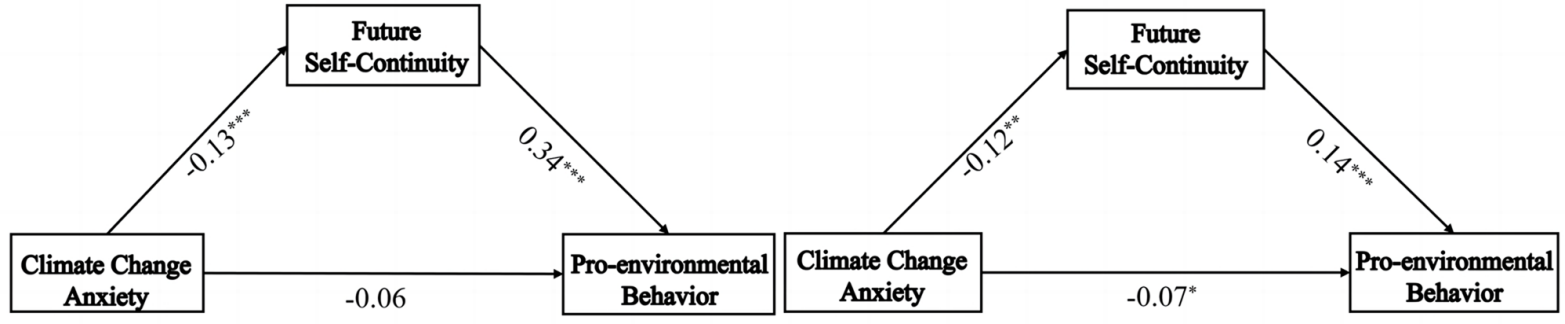
Mediating effect analysis

The data-set was created in SPSS 26.0 and the PROCESS macro program [81]. Model 4 in PROCESS was applied to test the mediating role of future self-continuity between middle school and high school levels, respectively. The results in Table 3 showed that, under the control of gender and age, climate change anxiety significantly predicted future self-continuity in both middle and high school levels (Middle: *β* = -0.13, *t* = -4.42, *p* < 0.001; High: *β* = -0.12, *t* = -3.46, *p* < 0.001). Climate change anxiety in middle school students could not significantly predict pro-environmental behavior (Middle: *β* = -0.06, *t* = -1.83, *p* > 0.05), and climate change anxiety in high school students could significantly predict pro-environmental behavior (High: *β* = -0.07, *t* = -2.00, *p* < 0.05). Future self-continuity significantly predicted pro-environmental behavior in both middle and high school levels (Middle: *β* = 0.34, *t* = 11.26, *p* < 0.001; High: *β* = 0.14, *t* = 4.25, *p* < 0.001). Mediation effects were significant at the middle and high school levels (Middle: indirect effect = -0.045, *SE* = 0.014, 95% CI = [-0.074, -0.020]; High: indirect effect = -0.017, *SE* = 0.009, 95% CI = [-0.037, − 0.003])(see Fig. 2). The above results supported hypothesis 1.

**Table 3:** Analysis of intermediation effects

Climate change anxiety	Middle school students						High school students				
	Model 1 (Future Self-Continuity)			Model 2 (Pro-environmental Behavior)			Model 1 (Future Self-Continuity)			Model 2 (Pro-environmental Behavior)	
	β	t		β	t		β	t		β	t
Constant	1.78	3.62^***^		1.26	2.69^**^		1.86	3.13^**^		1.43	2.40^*^
Age	-0.14	-3.62^***^		-0.10	-2.83^**^		-0.11	-3.04^**^		-0.09	-2.41^***^
Gender	-0.02	-0.27		0.12	2.04^*^		-0.08	-1.09		0.02	0.26
CCA	-0.13	-4.22^***^		-0.06	-1.83		-0.12	-3.46^***^		-0.07	-2.00^*^
FSC				0.34	11.26^***^					0.14	4.25^***^
*R* ^*2*^	0.04			0.14			0.28			0.38	
*F*	12.80^***^			41.44^***^			8.18^***^			8.33^***^	

Note.^*^p < 0.05; ^**^p < 0.01; ^***^p < 0.001

### Moderated mediation model test

We used Model 14 in the PROCESS macro program for testing moderated mediation. The results in Table 4 showed that, under the control of gender and age, climate change anxiety had a significant predictive effect on future self-continuity (Middle: *β*=-0.13, *t*=-4.22, *p* < 0.001; High: *β*=-0.12, *t* = 6.20, *p* < 0.001); and that future self-continuity significantly predicted pro-environmental behavior (Middle: *β* = 0.13, *t* = 4.68, *p* < 0.001; High: *β* = 0.02, *t* = 0.49, *p* < 0.001); Climate change anxiety could not significantly predict pro-environmental behavior (Middle: *β* = -0.02, *t* = -0.83, *p* > 0.05; High: *β* = -0.04, *t* = -1.40, *p* > 0.05);

The interaction between future self-continuity and green self-efficacy in middle school students positively predicted pro-environmental behavior (Middle: *β* = 0.07, *t* = 2.22, *p* < 0.05), while the interaction in high school students did not have a significant predictive effect (High: *β* = 0.05, *t* = 1.82, *p* > 0.05). The mediating effect of moderation was significant in middle school students (Middle: indirect effect = -0.008, *SE* = 0.004, 95% CI = [-0.017, -0.001]), but not significant in high school students (High: indirect effect = -0.005, *SE* = 0.004, 95% CI = [-0.016, 0.00]).

**Table 4:** Moderated mediation effect analysis

Climate change anxiety	Middle school students						High school students				
	Model 1 (Future Self-Continuity)			Model 2 ((Pro-environmental Behavior)			Model 1 (Future Self-Continuity)			Model 2 ((Pro-environmental Behavior)	
	β	t		β	t		β	t		β	t
Constant	1.78	3.62^***^		0.96	2.35^*^		1.86	3.13^***^		1.94	3.60^***^
Age	-0.14	-3.62^***^		-0.08	-2.54^*^		-0.11	1.39^**^		-0.12	-3.61^***^
Gender	-0.02	-0.27		0.10	1.95		-0.08	-0.38		-0.01	-0.17
CCA	-0.13	-4.22^***^		-0.02	-0.83		-0.12	6.20^***^		-0.04	-1.40
FSC				0.13	4.68^***^					0.02	0.49
GSE				-0.51	17.10^***^					0.45	14.35^***^
FSC × GSE				0.07	2.22^*^					0.05	1.82
*R* ^*2*^	0.02			0.35			0.03			0.23	
*F*	7.19^***^			86.69^***^			12.88^***^			41.89^***^	

Note.^*^p < 0.05; ^**^p < 0.01; ^***^p < 0.001

**Fig. 3:**
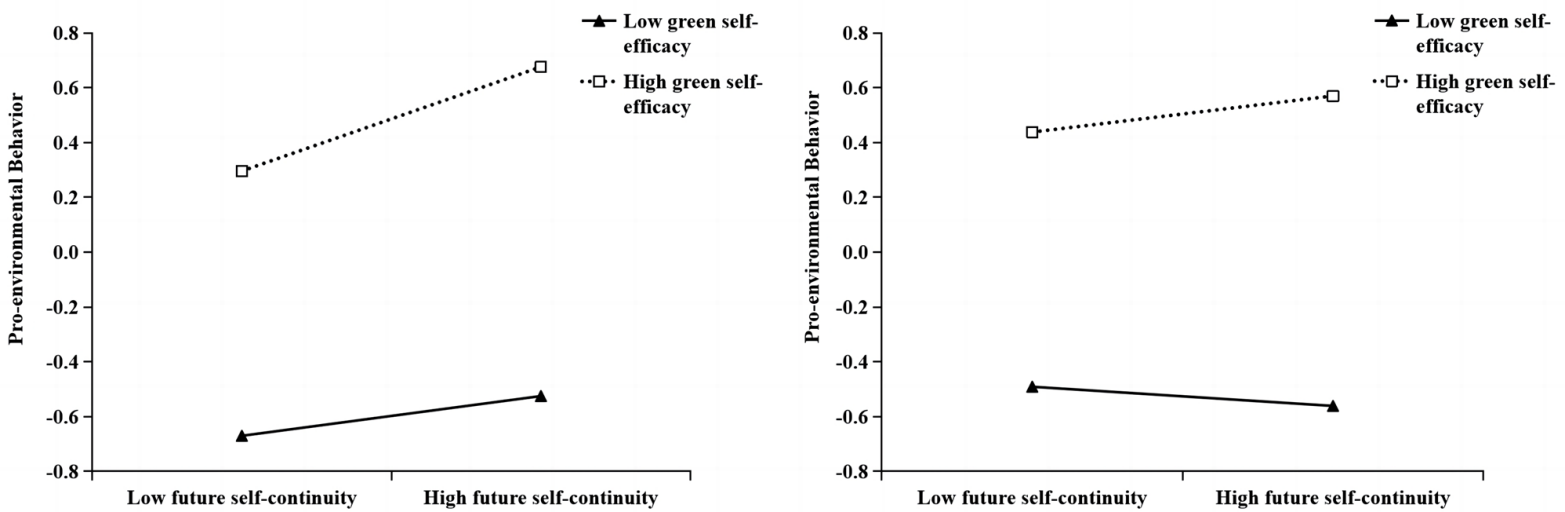
Simple slope analysis plot

To further explore the essence of the interaction between future self-continuity and green self-efficacy, this study used simple slope analysis to draw a simple effect map (see Fig. 3). It was found that in middle school level, future self-continuity did not significantly predict pro-environmental behaviors under low green self-efficacy level, and future self-continuity positively predicted pro-environmental behaviors under high green self-efficacy level, that was, the higher the future self-continuity, the more pro-environmental behaviors (low green self-efficacy group: *β* = 0.07, *t* = 1.74, *p* > 0.05, 95% CI = [-0.009,0.154]; high green self-efficacy group: *β* = 0.190, *t* = 5.149, *p* < 0.001, 95% CI = [0.118,0.263]). In high school level, whether at high or low levels of green self-efficacy, future self-continuity could not significantly predict pro-environmental behavior (Low green self-efficacy group: *β* =-0.036, *t* =-0.817, *p* > 0.05, 95%CI = [-0.121,0.050]; High green self-efficacy group: *β* = 0.067, *t* = 1.622, *p* > 0.05, 95%CI = [-0.014,0.149]).

**Fig. 4:**
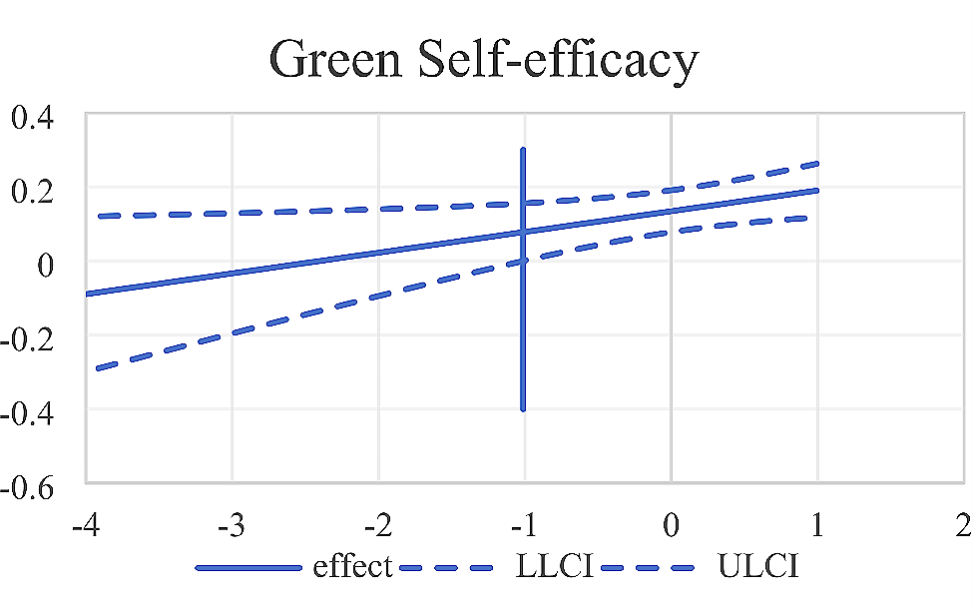
Moderating role of green self-efficacy

To further demonstrate the moderating role of green self-efficacy, this study was analyzed by using the Johnson-Neyman method [82]. It was found (as shown in Fig. 4) that in middle school, when green self-efficacy was higher than − 1.0121, the 95%CI of the simple slope didn’t include 0, and the simple slope was significant, indicating that future self-continuity had a significant predict on pro-environmental behavior; When green self-efficacy was lower than − 1.012, the 95%CI of the simple slope included 0, and the effect was not significant. Therefore, the moderating effect of green self-efficacy at the high school level was not present.

## Discussion

This study found that there was a negative correlation between climate change anxiety and adolescents’ pro-environmental behaviors, in which future self-continuity played a mediating role, and green self-efficacy moderated the pathway of adolescents’ climate change anxiety and pro-environmental behaviors.

In existing studies, there is controversy over the relationship between climate change anxiety and pro-environmental behavior, which suggest that climate change anxiety may promote or reduce pro-environmental behavior. However, this study found a significant negative correlation between climate change anxiety and pro-environmental behavior. That is, the more obvious the individual perceived climate change anxiety, the less inclined to make pro-environmental behavior. Consistent with the previous results, the research results indicate that excessive climate change anxiety inhibit people from translating their concerns into practical actions [27, 28]. Meanwhile, studies have also recognized that climate change anxiety will weaken individual response abilities, form defense mechanisms, make them feel indifferent and denied, and thus less inclined to engage in pro-environmental behaviors [73].

In the era of the Internet, various media often make comments about “turning points” and “temperature thresholds”. In fact, despite climate change, the likelihood of dying from weather disasters is decreasing, and with inflation and asset growth, the economic losses caused by weather disasters have not actually significantly increased. However, because teenagers have limited knowledge and skills about environmental change, it is easy to amplify their anxiety when they faced the threat posed by climate change. The Theory of Planned Behavior suggests that three factors, namely behavioral attitude, subjective norms, and perceived behavioral control, all have an impact on individual pro-environmental behavior [83]. The high levels of climate change anxiety affected cognitive control and executive function, and then decreased their intention to engage in more pro-environmental behaviors. That is to say, when adolescents receive a large amount of information about the threat of climate change, they will increase their level of anxiety. However, they tend to think that they are weak and difficult to take actions that are conducive to protecting the environment. Therefore, they prefer powerful individuals or institutions such as the government to protect the environment, so their attitude towards the environment becomes more indifferent and negative.

Previous research found that individuals with high future self-continuity will more richly imagine the future and make pro-environmental behaviors [49]. This study also found that future self-continuity not only was negatively correlated with climate change anxiety and positively correlated with pro-environmental behaviors, but played a mediating role between climate change anxiety and pro-environmental behaviors. That is, the higher the anxiety of climate change, the more individuals pay attention to the present; and the less attention to future self, the less willing they are to engage in pro-environmental behavior that benefits their offspring.

According to the theory of self-perception and self-determination [84, 85], an individual’s self-awareness is not only limited to the present, but also associated with their past and future. The stronger the psychological connection between the present self and future self, the easier it is to motivate individuals to make more visionary behaviors. However, when individuals have a stronger sense of anxiety about climate change, they tend to focus on present self and think less about future self, resulting in fewer pro-environmental behaviors [47, 48]. Based on explanatory level theory [86], the closer an individual felt the time distance, the more pro-environmental behaviors they engage in. Individuals with strong future self-continuity will perceive a closer time distance, and the connection between the future self and the present self will become closer, making them realize that current behavior will benefit the future and then more inclined to engage in more pro-environmental behaviors. Cultural orientation also plays a crucial role. Individualistic and collectivist orientations have been found to influence pro-environmental behaviors [87]. Related studies shared the same view that collectivists who care about group norms and collective harmony subordinate their individual goals to group goals and are more likely to engage in a variety of pro-environmental behaviors [88–90]. Chinese people are deeply influenced by collectivism, family consciousness, and Confucian ethics and morals. They tend to connect events that occurred in the past, present, and future as a whole in the temporal dimension, and believe that their ancestors, themselves, and their descendants are a complete continuation [91]. This is specifically reflected in the “big self” advocated in Chinese culture, which is completely different from the individualism advocated in Western culture. Under the influence of this cultural background, teenagers are more aware that the present and future are closely related as a whole, and the stronger the continuity of their future, the more willing they are to make pro- environmental behaviors.

In addition to the role of future self-continuity, green self-efficacy also plays as a moderator between climate change anxiety and pro-environmental behaviors. In this study, in the low green self-efficacy group, future self-continuity did not significantly predict environmental behaviors. This result indicates that individuals with low green self-efficacy, even if there is a psychological connection between their present and future selves, they cannot effectively engage in more environmentally friendly behaviors. This result confirms previous research that green self-efficacy can have an impact on pro-environmental behavior, so improving individual green self-efficacy can indirectly promote the implementation of pro-environmental behavior [53–56].

Moreover, the moderating effect of green self-efficacy only appeared in the group of middle school students. Previous study found the influence of different age on pro-environmental behavior [56]. According to social identity theory [92], group identity is an individual’s absolute obedience to a social unit. Individuals with group identity will internalize the rules of the group as their own behavioral norms, and the connotation of the group helps to form their self-concept. At the same time, the identity of group members will also drive their behavior. For high school students, pro-environmental behavior is more influenced by factors such as social and community environmental atmosphere. Due to the closer contact between high school students and the environment, they subconsciously believe that their actions belong to green environmental protection behavior, so they are not affected by their sense of efficacy when doing environmentally friendly behavior. Middle school students, on the other hand, due to their young age and lack of stable social identity, produce a strong sense of belonging. As a result, when the green self-efficacy was higher, middle school students recognized they should engage in pro-environmental behaviors.

### Conclusions and limitations

This study explained the underlying mechanism by which climate change anxiety affected pro-environmental behaviors. Climate change anxiety predicted future self-continuity, which in turn affects pro-environmental behaviors. It also found that green self-efficacy plays a moderating role in the relationship between future self-continuity and pro-environmental behaviors among middle school students.

This study has some limitations. First, this study used a cross-sectional survey study from which we could not draw causal conclusions. Second, the subjects in this study were all Chinese, and geographical and cultural differences were not considered. Some studies have shown that there are cross-cultural differences in the concept of future self-continuity. For example, the “I” in the Western definition of future self-continuity is the “small self”. In contrast, the “I” in Chinese future self-continuity should be the “big self” that includes significant others. The difference in conceptualization may lead to differences in the interpretation of the underlying mechanisms between climate change anxiety and pro-environmental behaviors. Future research could be conducted in more social contexts.

## Data Availability

The data that support the findings of this study are available from the corresponding author upon reasonable request.
